# Physicochemical properties of mountain streams in the High and Western Tatras

**DOI:** 10.1007/s10661-023-12158-w

**Published:** 2023-11-28

**Authors:** Jaroslav Solár, Jakub Tomaškovič

**Affiliations:** Institute of High Mountain Biology, University of Zilina, Tatranská Javorina, 7, SK-059 56, Tatranská Javorina, Zilina, Slovakia

**Keywords:** Water quality, Mountain streams, Physicochemical properties, Tatra Mountains, TANAP

## Abstract

The aim of this study was to measure the physicochemical properties of 28 mountain streams in Tatra National Park, Slovakia. Sampling sites (119) were selected based on a previous study conducted in 2010. Physical properties (e.g., temperature, conductivity, total dissolved solids, pH, and dissolved oxygen) and chemical components (e.g., nitrogen oxides, ammonia oxides, chloride compounds, and chemical oxygen demand) of the water were determined. Environmental parameters of streams (elevation, slope, aspect, width, depth, flow accumulation, watershed size, bedrock, and presence of mountain lakes) at sampling sites were examined. While comparing results from both periods (2010 and 2017), we found a correlation in data trends, concluding that elevation plays a significant role in almost all investigated parameters. Downstream, streams were more saturated by dissolved solids, CaCO_3_, and nitrates, increasing the pH level. Despite this well-known trend, we observed significant higher levels of ammonias and chlorides in the alpine zone, especially at sites where higher water temperature and lower values of dissolved oxygen were observed. This occurred in the Eastern Tatras, below mountain lakes, and where watersheds had granite origins. There are indications that denitrification processes were significantly stronger in 2017, but, on the other hand, increased chlorides resulted in stronger inhibition of nitrification processes in alpine zones at sites below mountain lakes.

## Introduction

Surface waters are an important part of mountain environments, and their quality reflects both natural and anthropogenic processes occurring within this system. There are many driving forces (natural and anthropogenic) that may affect the state of surface water in the short or long term. Each mountain environment is characterized by a high heterogeneity of conditions (such as topology, climate, geology, and biodiversity), and we can expect different states of surface water in relation to site conditions. It is accepted that mountains form a barrier to moving air masses, making this environment rich in precipitation, but also more susceptible to pollution deposition (particularly from long-range transmission) (Kopáček et al., 2006; Janiga & Haas, 2019; Janiga et al., 2019; Ballová et al., 2020). Changes to climate can affect the state of surface water in this complex high elevation environment. When we take into consideration that summer and winter temperatures have risen during the past 50 years (Melo et al., 2013), we can expect changes in surface water quantity, including the timing of snow melt and summer low flows, as well as changes in surface water quality (Kopáček et al., 2019; Paillex et al., 2020).

The Tatra Mountains are a unique mountain system located in central Europe and create one of the major European watershed divides. For natural scientists, the Tatras have always been an attractive locale to study lakes and streams. The first researchers began collecting and publishing data on flora and fauna of Tatra waters more than a century ago (Nowicki, 1868; Wierzejski, 1881). In the 1960s and 1970s, many complex limnological projects were conducted in the Tatras (e.g., Juriš et al., 1965; Kownacka & Kownacki, 1965; Kownacki, 1977), resulting in the first comprehensive data on chemistry, biota, and ecology of mountain rivers (Ertl, 1984). At the time, research was also motivated by concerns regarding the deterioration of water quality with increasing tourism. The issue of acidification of the environment also came to the fore in the 1980s. Stuchlík et al. (1985a, b) evaluated a set of 268 sites in 1981–1983, comparing them with older data (Ertl, 1965; Ertl et al., 1965; Juriš et al., 1965; Stangenberg, 1938; Bombówna, 1965), and found significant increases in NO_−3_ and SO_2_^−4^ concentrations, which indicated that acidification was mainly due to ions of strong acids (sulfuric and nitric) from precipitation. Later, Kopáček et al. (2006) analyzed and compared those results with data (only from mountain lakes) from 1994 and 2004, and they noted that concentrations of SO_2_^−4^ and NO_−3_ have decreased by ∼50% on average since 1984. This recovery effect was also confirmed in European surface waters (Wright et al. 2005; Battarbee et al. 2014; Borg & Sundbom 2014). In contrast, results from high mountain streams, where glaciers are present, did not show decreases in SO_2_^−4^ and NO_−3_, due to the melting of glaciers (Baron et al., 2009). Recent studies from the Tatra region confirm that water temperature has increased 0.3°C from 1971 to 2015 (Ptak et al., 2017); however, the amount of that increase was dependant on weather conditions, catchment characteristics, and flow rate (Żelazny et al., 2018). Seasonal changes in the concentration of basic elements in water are dependent on geological structure (Sajdak et al., 2018), mean flow, and flow rate and correlate with precipitation, especially on open and steep slopes (Radecki-Pawlik et al., 2020). Ongoing changes in climate, such as increasing annual air temperature, especially in April and August (Zeleňáková et al. 2018a, b), or shifting of vertical climatic belts (Łupikasza and Szypuła 2019), affect chemistry and composition of water through an increase of rock weathering and leaching of nutrients from soil (Kopáček et al., 2017; Kopáček et al., 2019).

Tourism may also be affecting water quality in the region. Sobczyk et al. (2020) noted the potentially large ecological impact of mass uncontrolled tourism on the region. Tourism development and visitation of the mountains has increased in recent years (Getzner & Švajda 2015). These activities can increase the probability of soil erosion and subsequently flush nutrients from eroded areas into streams. Dynowski et al. (2019) confirm that trails in the vicinity of alpine lakes contribute to the growth of aquatic vegetation in lake bottoms. Additionally, it has been shown that excessive tourism leads to higher wastewater production from tourist accommodations, and the use of undesignated toilets (close to tourist paths), which are frequently partnered with other non-decomposable forms of waste.

In the 1980s, the Research Base for Mountain Hydrology under the body of The Institute of Hydrology (Slovak Academy of Science) published a series of studies on mountain river networks, water quality, and its further development in high mountain regions. However, additional studies also describe the physicochemical properties of mountain lakes in the region. Studies of mountain streams are typically more focused on individual streams or small areas. One exception was a study in 2010 in which Gura et al. (2013) described the physicochemical properties of 119 sampling locations across 28 streams in the Slovak part of the Tatra Mountains. There was an inverse relationship between nitrate concentration and altitude as well as between conductivity and altitude. This study returned to sample those same 119 locations in 2017 to investigate changes in the streams’ physicochemical properties and the drivers of that change.

## Material and methods

### Study area description

Research aimed at selected mountain streams is ongoing in the Slovak part of the Tatra Mountains (established in 1949 as Tatra National Park), which are the highest-altitude mountain range in the Carpathians. The area is characterized by well-preserved high mountain ecosystems with wide biodiversity. From a geological standpoint, the area is dominantly comprised of granitic bedrock, followed by metamorphic rocks, gneiss, mica schist, and limestone with dolomites (Nemčok et al., 1993). Cold climate is dominant at higher elevations, with an average temperature of < 10 °C in June. Lower elevation sites in sub-mountainous areas have average temperatures of 12–16 °C. Annual precipitation, according to elevation, ranges from 600 to 2000 mm (Tomlain, 2002). Mountain streams belong to the catchment basins of both the Black Sea (via Váh–Danube) and Baltic Sea (via Poprad–Dunajec–Vizsla).

### Sample collection methods

**Fig. 1:**
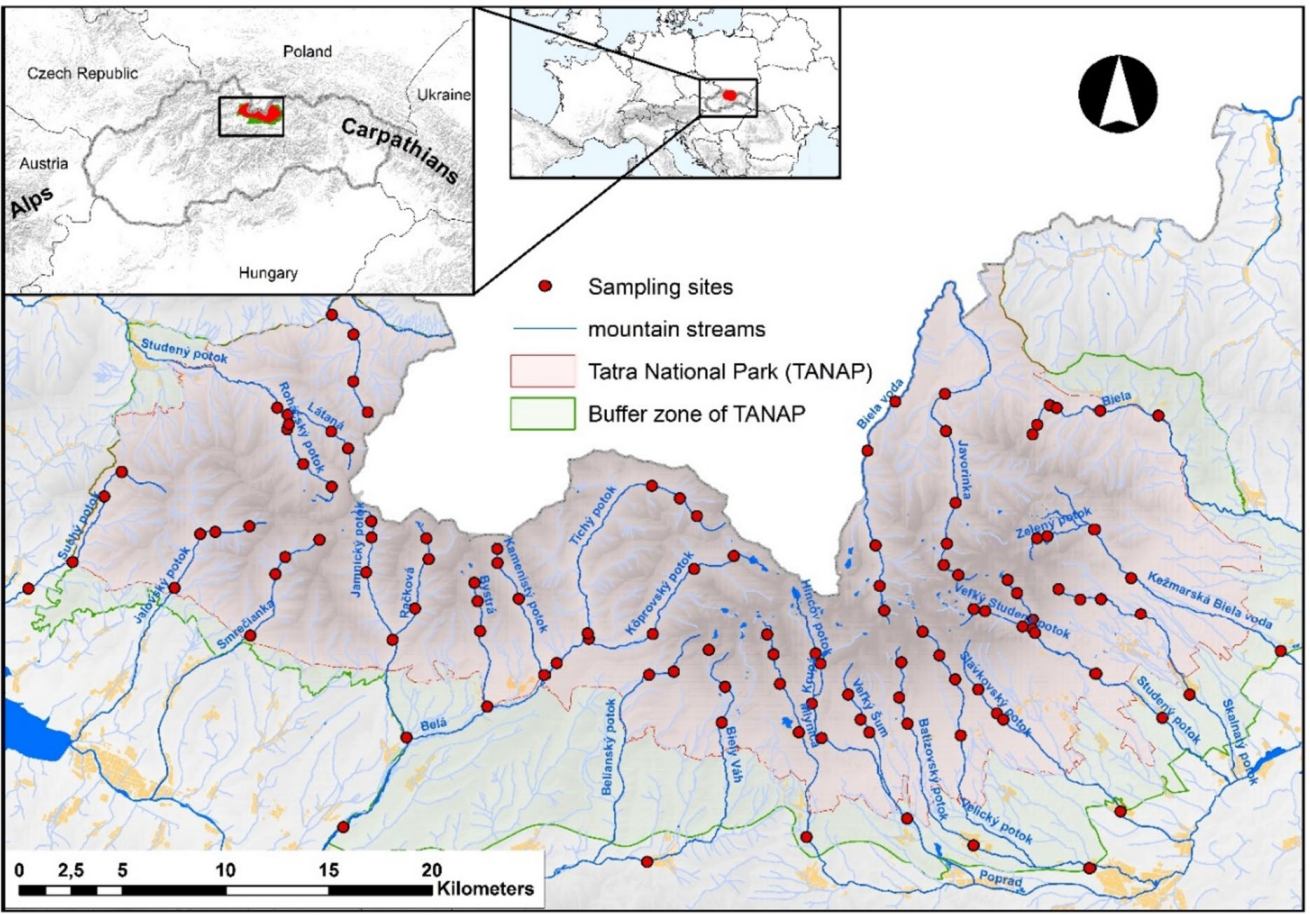
Tatra National Park (Slovakia) with its main streams and the sampling sites of data collection

Sampling was carried out during the growing season (between June and September) in 2017. In 2010, Gura et al. (2013) monitored 28 streams in TANAP, which represented the main mountain streams from almost all valleys in the Tatra Mountains (Fig. 1). We used the same sampling locations as Gura et al. (2013) and sampled during the same period of the year (June through October) to compare changes in stream water chemistry between the two study periods. Samples were taken from four to six places along an altitudinal gradient from the submontane to the alpine region, 639–2002 m a.s.l. In the east part of the Tatra Mountains, the following streams were monitored: Biela voda, Javorinka, Biela. Kežmarská Biela voda, Skalnatý potok, Malý Studený potok, and Veľký Studený potok, which forms Studený potok, Slavkovský potok, Velický potok, Batizovský potok, Veľký Šum, Poprad, Mlynica, Biely Váh, and Belianský potok. In the west part of the Tatra Mountains, the following streams were monitored: Tichý potok and Kôprovský potok (which form the Belá), Kamenistý potok, Bystrá, Račkov potok, Jamnický potok, Smrečianka, Jalovecký potok, Suchý potok, Látana, Roháčsky potok, Studený potok, and Bobrovecký potok. GPS (WGS84) determined each sampling site according to coordinates from the study by Gura et al. (2013). Days for sample collection were precisely chosen based on the timing of heavy rain. Each field day was selected no earlier than 3 days following heavy rain at the site. In total, 119 samples from streams were taken in the study area. During sampling, environmental conditions such as air temperature (°C), slope gradient (°), elevation (m a.s.l.), width and depth (m) of the water body, eventual negative influences, sources of pollution, and descriptions of the shore and bottom of riverbeds were recorded in the field protocol. Mean air temperature and cumulative precipitation for individual months during sample collection are presented in Table 1.

**Table 1:** Mean air temperature and cumulative amount of precipitation for individual months during sample collection. Values from meteorological station of Slovak Hydrometeorological Institute at Lomnický peak (2634 m a.s.l.) in Tatra Mountains

	June	July	August	September	
Mean air temperature in 2010 (°C)	4.9	6.9	6.0	0.2	Mean 4.5
Mean air temperature in 2017 (°C)	4.5	4.8	7.2	0.6	Mean 4.3
Cumulative amount of precipitation in 2010 (mm)	154.6	305.0	150.5	215.2	Sum 825.3
Cumulative amount of precipitation in 2017 (mm)	115.7	171.3	136.8	181.2	Sum 605.0

### Water analyses

In situ, physicochemical parameters such as water temperature (in °C), pH, conductivity (COND in μS), total dissolved solids (TDS in mg/L), dissolved oxygen content (DO in mg/L), and dissolved oxygen saturation (DO_%_ in %) were repeated three times and measured by a Multi device 3430 (WTW GmbH, Weilheim, Germany). Resultant values were averaged. Samples for laboratory analysis were taken in sterile glass laboratory bottles (1000 ml) and stored in a bag with frozen gel bars. Consequently (to 12 h), following field work, the chemical properties of water samples were measured in the laboratory. Samples were not preserved and filtered. We analyzed nitrate (NO_3_ as N in mg/L), ammonia (NH_3_ and NH_4_ as N, in mg/L), and chlorides (Cl and NaCl in mg/L) using the YSI 9500 Photometer (YSI Inc., Ohio, USA) based on reagents, and chemical oxygen demand (COD in mg/L) using manganometric titration according to the standardized procedure by Horáková et al. (1989). All samples were tested three times and averaged with relative standard deviation below 10%. Before each measurement (samples and chemical values), the photometer was calibrated using a blank sample.

### Statistical analyses

Statistical evaluation was performed in Statistica 12 (StatSoft, USA). A single data matrix was created from both datasets (2010 and 2017) and consequently evaluated using various statistical techniques. During validation, some lower-altitude sites were excluded due to inconsistent timing of samples, as Gura et al. (2013) collected some samples at the lowest elevation sites of streams in late October, while we collected those samples during stream examination. According to the Shapiro-Wilk normality test, the data did not have a normal distribution, except for elevation, air, water temperature, and DO. Therefore, a non-parametric approach to the analysis of the data was necessary. The Spearman’s rank correlation coefficient was used for assessment of mutual relationships of variables, and the Mann-Whitney *U* test was used to compare the medians of the physicochemical variables with categorical variables, which only have two groups (true or false). Variables with more than two groups were tested using the Kruskal-Wallis test H (ANOVA by Ranks). Principal component analysis (PCA) was used to extract the potential relationships between the joint variables measured in 2010 and 2017. Before PCA data were normalized, resulting principal components (factor coordinates of cases) with eigenvalue greater than one were tested by ANOVA with categorical variables, such as the year of sampling (2010, 2017), vegetation zones (alpine, sub-alpine, forest, deforested, and developed areas), sampling sites with possible mountain lake effect (true, false), and base rock (igneous, sedimentary, and metamorphic rocks).

## Results

Joint descriptive statistics for all sites and both time periods (2010 and 2017) show that the air and water temperature were higher in 2010, as well as values of pH and dissolved oxygen content (DO). On the other hand, total dissolved solids (TDS), conductivity (COND), dissolved oxygen saturation (DO_%_), chemical oxygen demand (COD), total hardness (CaCO_3_), and concentrations of ammonia, nitrates, and chlorides were lower in 2010. Significant differences were achieved in the case of TDS, COND, DO, DO_%_, COD, nitrates, and chlorides (Table 2). Water temperature had a significant negative correlation with DO, and a significant positive correlation with ammonia in both periods (Table 3). Though mean water temperature was lower in 2017, it also had a significant positive correlation with concentrations of nitrates and chlorides, and a negative correlation with pH and TDS levels. Higher pH values were likely attributable to TDS and CaCO_3_ in water, as well as by nitrates, specifically in 2010. In 2017, we can see that pH values were more influenced by TDS than by CaCO_3_, and pH values were additionally negative correlated with water temperature and higher concentrations of ammonias and chlorides. That led us to the assumption that pH level in 2017 decreased due to a unique way of weathering or leaching of substances from soils or bedrocks driven by hydroclimatic factors. The negative correlations of DO with concentrations of ammonias, nitrates, and chlorides can also be explained by this phenomenon in 2017.

**Table 2:** Differences of physicochemical parameters measured in water samples collected from mountain streams in the Slovakian Tatra Mountains in 2010 and 2017. Computed by Mann-Whitney or Kruskal-Wallis tests (*p* < 0.05 are significant differences; groups–years: a 2010, b 2017; Tatras: *E* Eastern, *W* Western Tatra Mountains; Geology: *I* igneous, *S* sedimentary, *M* metamorphic rocks; Zones: 1, alpine; 2, sub-alpine; 3, forest; 4, deforested; 5, developed areas; Lakes: 0, highest 2 sites at each stream without mountain lakes; 1, highest 2 sites at each stream with mountain lakes to a distance of 1 km; bolded *p* values are significant for all data from both time periods)

Variables	Years		Tatras		Geology		Zones		Lakes	
Air temp (°C)	0.508		**0.001**^**b**^	**E > W**	**0.039**^**ab**^	**I > M > S**	**0.030**^**a**^	**14253**	0.063	
H2O temp (°C)	0.095	a > b	**0.001**^**b**^	**E > W**	0.877^b^	I > S > M	0.122^a^	54321	0.504	
pH	0.089	a > b	**0.001**^**ab**^	**W > E**	**0.001**^**ab**^	**S > M > I**	**0.001**^**ab**^	**53421**	**0.001**^**ab**^	**0 > 1**
COND (μS)	**0.001**	**b > a**	**0.001**^**ab**^	**W > E**	**0.001**^**ab**^	**S > M > I**	**0.001**^**ab**^	**53421**	**0.001**^**ab**^	**0 > 1**
TDS (mg/L)	**0.001**	**b > a**	**0.001**^**ab**^	**W > E**	**0.001**^**ab**^	**S > M > I**	**0.001**^**ab**^	**53421**	**0.001**^**ab**^	**0 > 1**
O_2_ (mg/L)	**0.001**	**a > b**	**0.001**^**ab**^	**W > E**	0.327^b^	M > S > I	0.332		**0.011**^**b**^	**0 > 1**
O_2_ (%)	**0.001**	**b > a**	**0.001**^**a**^	**W > E**	0.718^b^	I > M > S	0.551^ab^		0.206^a^	
COD (mg/L)	**0.000**	**b > a**	0.842		0.565		0.184		0.230	
CaCO_3_ (mg/L)	0.056		**0.001**^**ab**^	**W > E**	**0.001**^**ab**^	**S > M > I**	**0.001**^**a**^	**53421**	**0.001**^**ab**^	**0 > 1**
Nitrate N (mg/L)	**0.001**	**b > a**	**0.024**^**b**^	**E > W**	**0.002**^**a**^	**S > I > M**	**0.005**^**ab**^	Fig. 2A	0.227^b^	1 > 0
Nitrate NO_3_ (mg/L)	**0.001**	**b > a**	**0.049**^**b**^	**E > W**	**0.002**^**a**^	**S > I > M**	**0.013**^**ab**^		0.104^b^	1 > 0
Ammonia N (mg/L)	0.799		**0.001**^**ab**^	**E > W**	0.591^b^	I > S > M	0.437		**0.029**^**b**^	**1 > 0**
Ammonia NH_3_ (mg/L)	0.799		**0.001**^**ab**^	**E > W**	0.589^b^	I > S > M	0.472		**0.032**^**b**^	**1 > 0**
Ammonia NH_4_ (mg/L)	0.773		**0.001**^**ab**^	**E > W**	0.583^b^	I > S > M	0.460		**0.032**^**b**^	**1 > 0**
Chloride Cl (mg/L)	**0.001**	**b > a**	**0.001**^**b**^	**E > W**	**0.001**^**ab**^	**I > S > M**	**0.002**^**ab**^		**0.023**	**1 > 0**
Chloride NaCl (mg/L)	**0.001**	**b > a**	**0.001**^**b**^	**E > W**	**0.001**^**ab**^	**I > S > M**	**0.002**^**ab**^	Fig. 2B	**0.022**	**1 > 0**

**Table 3:** Spearman correlation coefficient by rank for measured variables of water samples collected from mountain streams in the Slovakian Tatra Mountains (mutual correlation coefficients for years 2010 and 2017 are in frame; significant correlations are in bold; correlation coefficients of 2017 are in italic)

	T_Air_	T_H2O_	pH	COND	TDS	DO	DO_%_	COD	CaCO_3_	N	NO_3_	N	NH_3_	NH_4_	Cl	NaCl
T_Air_	0.06	***0.75***	−***0.56***	−***0.42***	−***0.42***	−***0.75***	−*0.03*	*0.02*	−*0.22*	***0.41***	***0.36***	***0.59***	***0.59***	***0.59***	***0.60***	***0.60***
T_H2O_	**0.62**	**0.20**	−***0.46***	−***0.32***	−***0.32***	−***0.87***	−***0.31***	*0.05*	−*0.13*	***0.43***	***0.43***	***0.49***	***0.49***	***0.49***	***0.55***	***0.55***
pH	−0.01	0.15	**0.73**	***0.91***	***0.91***	***0.59***	−*0.22*	−*0.12*	***0.55***	−*0.21*	−*0.21*	−***0.39***	−***0.39***	−***0.38***	−***0.43***	−***0.43***
COND	0.04	0.18	**0.79**	**0.93**	***0.99***	***0.52***	−***0.28***	−*0.21*	***0.58***	−*0.12*	−*0.12*	−***0.29***	−***0.29***	−***0.29***	−***0.35***	−***0.35***
TDS	−0.02	0.12	**0.82**	**0.99**	**0.95**	***0.51***	−***0.29***	−*0.21*	***0.58***	−*0.12*	−*0.12*	−***0.29***	−***0.29***	−***0.29***	−***0.35***	−***0.35***
DO	−**0.54**	−**0.71**	0.20	0.01	0.06	**0.27**	*0.09*	−*0.03*	***0.29***	−***0.38***	−***0.41***	−***0.49***	−***0.49***	−***0.48***	−***0.52***	−***0.52***
DO_%_	−**0.24**	−0.17	**0.41**	0.18	0.20	**0.81**	−0.17	*0.07*	−*0.16*	−*0.18*	−*0.19*	*0.12*	*0.12*	*0.13*	*0.10*	*0.10*
COD	0.08	**0.38**	0.22	0.19	0.19	−0.09	0.18	**0.34**	*0.00*	−*0.11*	−*0.20*	−*0.01*	*0.00*	−*0.01*	*0.14*	*0.14*
CaCO_3_	−0.02	0.16	**0.82**	**0.99**	**0.99**	0.03	0.18	0.20	**0.59**	−*0.02*	−*0.02*	*0.00*	*0.00*	*0.00*	−*0.04*	−*0.04*
Nit. N	−0.09	0.04	**0.57**	**0.73**	**0.74**	0.14	0.22	0.07	**0.73**	**0.39**	***0.91***	***0.46***	***0.46***	***0.45***	*0.20*	*0.20*
NO_3_	−0.09	0.04	**0.57**	**0.73**	**0.74**	0.14	0.22	0.07	**0.73**	**0.99**	**0.36**	***0.37***	***0.37***	***0.37***	*0.09*	*0.09*
Amo. N	0.05	0.09	−0.01	0.10	0.08	−0.09	−0.07	0.05	0.08	0.05	0.05	−0.09	***0.99***	***0.99***	***0.54***	***0.54***
NH_3_	0.05	0.09	−0.02	0.09	0.07	−0.09	−0.07	0.05	0.08	0.04	0.04	**0.99**	−0.09	***0.99***	***0.54***	***0.54***
NH_4_	0.05	0.10	−0.01	0.10	0.08	−0.10	−0.07	0.07	0.08	0.05	0.05	**0.99**	**0.99**	−0.09	***0.54***	***0.54***
Cl	0.02	0.10	−0.02	0.19	0.16	−0.08	−0.02	0.11	0.16	**0.30**	**0.30**	**0.40**	**0.40**	**0.41**	0.13	***0.99***
NaCl	0.02	0.10	−0.01	0.19	0.17	−0.09	−0.03	0.11	0.17	**0.30**	**0.30**	**0.41**	**0.41**	**0.41**	**0.99**	0.13

The Tatra Mountains are divided between the Eastern and Western Tatra Mountains, and we found differences between them, especially in samples collected in 2017. In the Western Tatras, we observed higher values of pH, TDS, and CaCO_3_ during both periods (Table 2). In the Eastern Tatras, we observed higher values of ammonias, and in 2017, we also observed higher values of nitrates and chlorides along with water temperature. These differences were partly influenced by bedrocks, as the Western Tatras are primarily comprised of metamorphic (Paleozoic) and sedimentary rocks (Mesozoic), while Eastern Tatras are dominated by granitic rocks. Therefore, chlorides were significantly higher in sites with granites. Similarly, water temperatures with ammonias were higher in those locations, but only in 2017. Nitrates were significantly higher in streams where sedimentary rock was present, particularly in 2010.

**Fig. 2:**
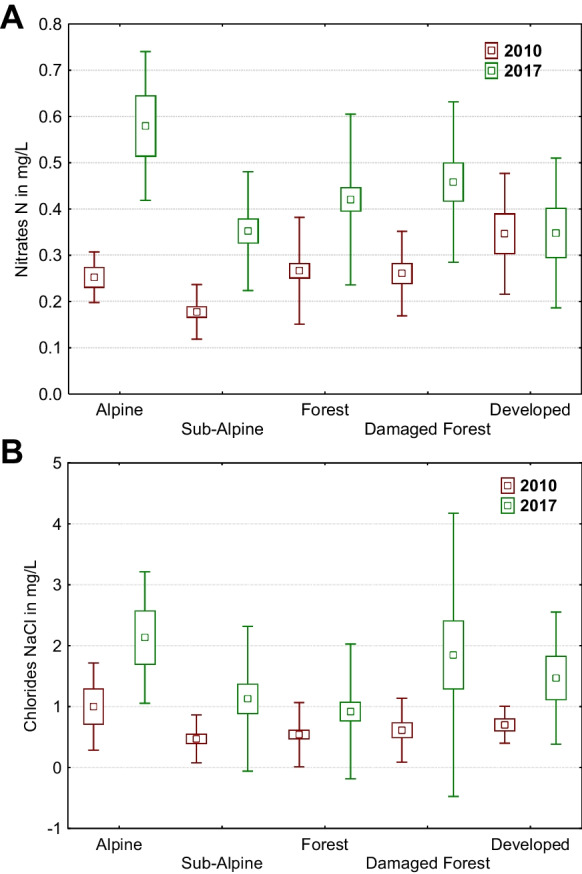
Differences between measured values of nitrates N (**A**) and chlorides NaCl (**B**) in each year (2010 and 2017) according to vegetation zones. Nitrates N: 2010 [K-W H (4, 105) = 26.718, *p* = 0.001], 2017 [K-W H (4, 105) = 11.106, *p* = 0.025]; chlorides NaCl: 2010 [K-W H (4, 105) = 8.224, *p* = 0.0837], 2017 [K-W H (4, 105) = 11.383, *p* = 0.023]; squares, means; box, standard errors of mean; whisker, standard deviations of mean

Differences in some measured values according to vegetation zones pointed to general trends applicable in both periods. The levels of pH, TDS, and CaCO_3_ were the highest in urban areas, followed by forested areas, with the lowest values observed in alpine and sub-alpine areas. Conversely, nitrates had the highest values in urban areas in 2010 but had the highest values in 2017 at alpine sites (Fig. 2A). The lowest values of nitrates were measured in the sub-alpine zone during both periods. Similarly, chlorides had different trends but the highest values were present in the alpine zone during both periods (this was only significant in 2017), and lower values were measured in sub-alpine zones and forested areas (Fig. 2B).

We assessed how mountain lakes (tarns) influence water chemistry and found significantly lower pH levels, TDS, and CaCO_3_ in samples collected below alpine lakes (up to 1 km) during both periods. On the other hand, we found significantly higher values of nitrates and ammonias in the data from 2017. Chlorides were also significantly higher in samples collected below mountain lakes, though the variation found between individual years was not significant. During our survey, we examined parameters of streams in sampling sites. These parameters were partly extended by GIS analysis (watershed size and flow accumulation) and resultant variables were correlated with physicochemical parameters measured in water samples (Table 4). As we expected, elevation plays a significant role in all investigated parameters, except for chlorides. Nitrates and COD were only significantly correlated in 2010. Higher nitrate concentrations were associated with wider streams with a small slope at a lower altitude, where water accumulation is greater. Ammonias were positively correlated with elevation, particularly in data from 2017.

**Table 4:** Correlation coefficients for physicochemical parameters and site parameters of streams which were measured in mountain streams in the Slovakian Tatra Mountains (significant correlations for joined data of both time period are in bold; indexed values pointed to significance only for one of the years; a 2010, b 2017; if the coefficient was not significant for joined data, it was replaced by a significant coefficient for the appropriate year

	Elevation (m a.s.l.)	Flow width (m)	Flow depth (m)	Flow slope (°)	Flow accumulation	Watershed size (m^2^)
Air temp (°C)	**0.14**	**−**0.20^a^	**−**0.27^a^	**−**0.07	**−**0.14	**−0.15**^**b**^
H2O temp (°C)	**−0.16**	0.24^b^	**−**0.01	**−0.26**	0.06	0.01
pH	**−0.60**	0.09	**0.23**	**−0.22**^**a**^	**0.19**^**b**^	**0.19**^**b**^
COND (μS)	**−0.49**	0.05	**0.14**	**−0.17**	0.12	**0.16**
TDS (mg/L)	**−0.48**	0.05	**0.15**	**−0.16**	0.11	**0.15**
O_2_ (mg/L)	**−0.21**	0.02	**0.14**^**a**^	0.01	0.13	0.08
O_2_ (%)	**−0.32**	0.06	**0.15**^**a**^	**−0.15**	**0.21**^**a**^	**0.15**^**a**^
COD (mg/L)	**−**0.31^a^	**−**0.20^b^	**−**0.04	**−**0.01	0.01	**−**0.06
CaCO_3_ (mg/L)	**−0.49**	0.04	**0.14**	**−0.16**	0.09	**0.14**
Nitrate N (mg/L)	**−**0.48^a^	**0.16**	0.10	**−0.20**^**a**^	0.26^a^	0.20^a^
Nitrate NO_3_ (mg/L)	**−**0.48^a^	**0.16**	0.12	**−0.22**^**a**^	0.26^a^	0.20^a^
Ammonia N (mg/L)	**0.15**^**b**^	**−**0.06	**−**0.04	**−**0.01	**−**0.12	**−0.14**
Ammonia NH_3_ (mg/L)	**0.15**^**b**^	**−**0.06	**−**0.05	**−**0.01	**−**0.13	**−0.14**
Ammonia NH_4_ (mg/L)	**0.14**^**b**^	**−**0.06	**−**0.05	**−**0.01	**−**0.13	**−0.14**
Chloride Cl (mg/L)	0.08	**−**0.02	**−**0.03	**−**0.04	**−**0.06	**−**0.07
Chloride NaCl (mg/L)	0.09	**−**0.02	**−**0.03	**−**0.04	**−**0.06	**−**0.07

**Fig. 3:**
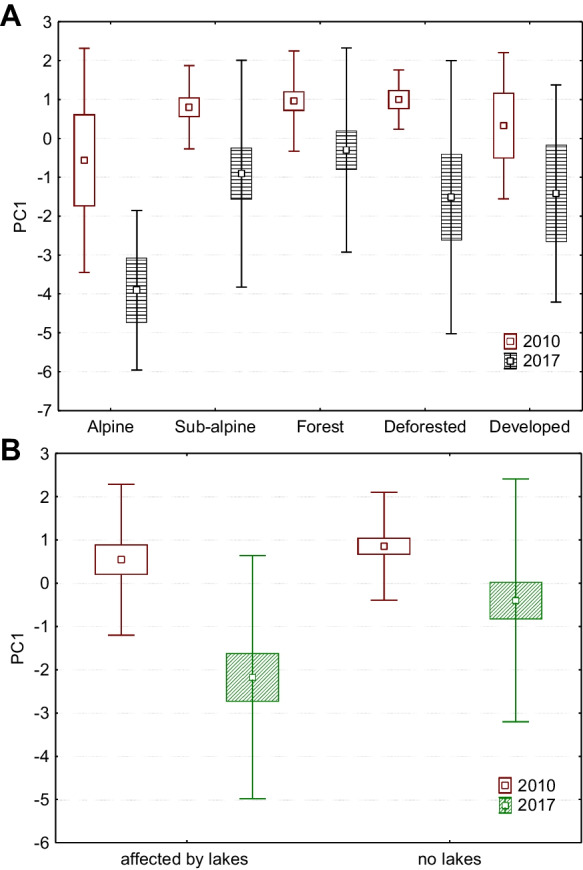
The first principal component represents higher levels of nitrates, ammonias, and chlorides at warmer sites with lower dissolved oxygen content. **A** With respect to vegetation zones [2010: F (4, 62) = 1.628, *p* = 0.179; 2017: F (4, 62) = 2.104, *p* = 0.091]. **B** With respect to lake influence [2010: F (1, 65) = 0.721, *p* = 0.399; 2017: our F (1, 65) = 6.257, *p* = 0.015]. Squares, means; box, standard errors of mean; whisker, standard deviations of mean

**Fig. 4:**
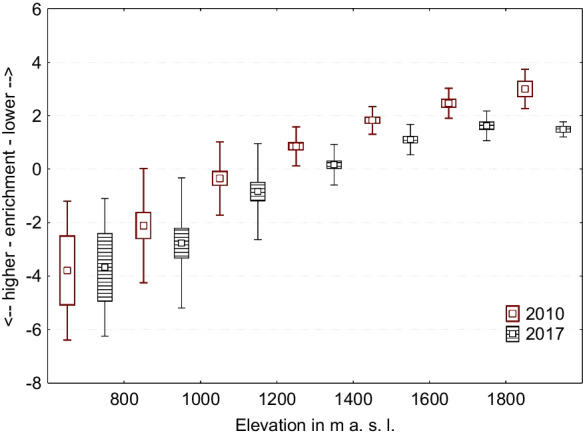
The second principal component is general water enrichment by basic chemical compounds with a strong relation to elevation. [2010: F (6, 98) = 32.295, *p* = 0.001; 2017: F (6, 98) = 19.473, *p* = 0.001; mutual effect of elevations and years: F (6, 196) = 0.342, *p* = 0.914]. Squares, means; box, standard errors of mean; whisker, standard deviations of mean

**Fig. 5:**
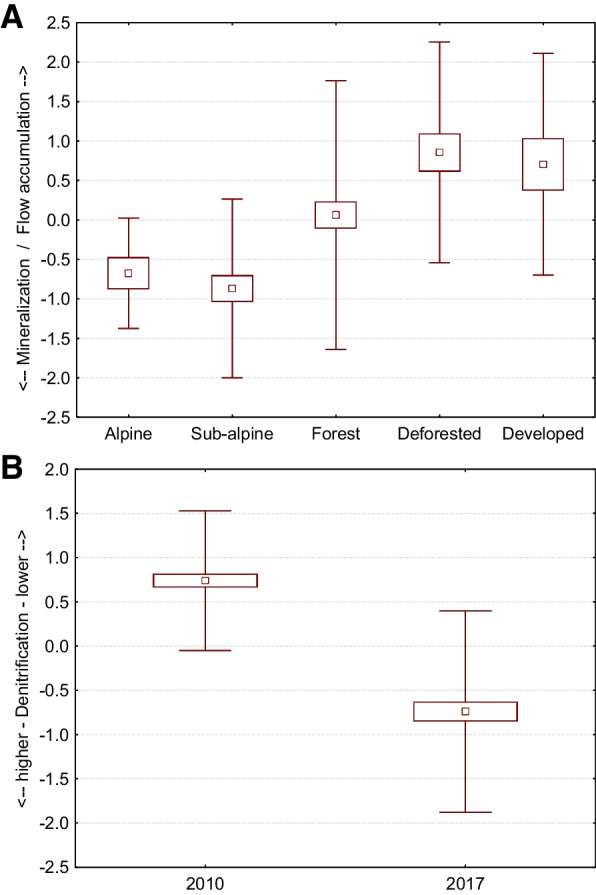
**A** The third component explains the level of mineralization (TDS and CaCO_3_) of the water in relation to flow accumulation [F (4, 205) = 8.504; *p* = 0.001]. **B** The fifth component is related to denitrification processes [F (1, 208) = 119.752, *p* = 0.001]. Squares, means; box, standard errors of mean; whisker, standard deviations of mean

**Fig. 6:**
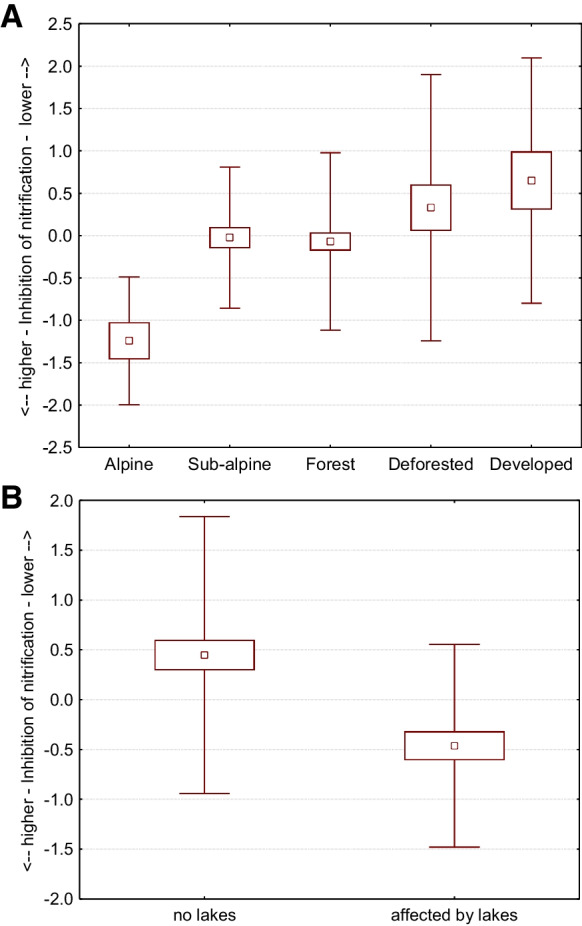
The sixth component is related to chemical reduction of nitrates by chlorides. **A** With respect to vegetation zones [F (4, 205) = 5.897, *p* = 0.001]. **B** With respect to lake influence [F (1, 132) = 16.288, *p* = 0.001]. Squares, means; box, standard errors of mean; whisker, standard deviations of mean

Looking back at the mutual correlation of physicochemical parameters (2010 vs. 2017), we can see that water temperature, pH level, COND, TDS, DO, COD, and concentration of nitrates had significant positive correlations (Table 3—in frames). These correlations show that the variables have a common trend (in both periods) and can be connected. It also confirms that this is not a random phenomenon. On the other hand, variables that did not correlate with each other point to a notable change in the trend, in the level of ammonias and chlorides compared to data from 2010. For better detection of mutual interaction of variables and ongoing trends, we used principal component analysis. We identified seven components with higher eigenvalues (over one) that together comprised 82% of data (Table 5). The first component (22.3%) represented higher levels of nitrates, ammonias, and chlorides at sites where water temperature was high and values of DO were low. This component is not influenced by elevation. However, we found significant differences, particularly in alpine environments (*p* = 0.0306). The comparison between years shows that 2017 was crucial for this component (Fig. 3A), and the presence of mountain lakes can significantly influence higher levels of nitrates, ammonias, and chlorides in streams of the alpine zone (Fig. 3B). The second component represented general water enrichment by basic chemical compounds with a strong relation to elevation (Fig. 4). Downstream samples were more saturated with TDS, CaCO_3_, and nitrates, which increased their pH level. Characteristic properties of streams changed farther downstream. Streams were wider, deeper, less steep, and took on higher values of water accumulation as the overall watershed increased. The third component is closely related to flow parameters of streams and their levels of TDS and CaCO_3_. This means that, in contrast to the second component, the colder, narrower, and steeper streams with lower flow accumulation had higher TDS and CaCO_3_ content in alpine and sub-alpine zones. On the other hand, warmer and wider streams with higher flow accumulation had lower values of TDS and CaCO_3_, predominantly in deforested and developed areas (Fig. 5A). The fourth component pointed to a mutual relationship of water temperature with oxygen and ammonia content in streams. This component is not dependant on elevation, but is characteristic of colder sites where ammonia accumulates, and ammonia-oxidizing processes driven by bacteria were slower. This finding supports several studies as Baron et al., (2009) or Clark et al. (2021). The fifth component represented the mutual interaction of COD and nitrates with the content of ammonia in streams. This means that values of COD are related to denitrification processes, which were significantly stronger in 2017 (Fig. 5B). The sixth component describes the mutual interaction of nitrates and chlorides, resulting in the inhibition of nitrification with accordance to study of Szklarek et al. (2022). This inhibition is influenced by the presence of mountain lakes, particularly in alpine zones (Fig. 6A and B). The seventh component explained the lack of oxygen saturated water samples in colder, higher-elevation sites, where streams were wider and with a steeper slope.

**Table 5:** Principal components and percent of variance associated with the components indicating seven main phenomena in mountain streams (for joint variables from both datasets 2010 and 2017)

**Variables**	**PC1**	**PC2**	**PC3**	**PC4**	**PC5**	**PC6**	**PC7**
Elevation (m a.s.l.)	**−**0.183	**0.753**	**−**0.223	0.014	**−**0.026	**−**0.261	0.236
Flow width (m)	0.090	**−**0.359	**0.646**	0.067	0.152	**−**0.209	0.287
Flow depth (m)	0.114	**−**0.409	0.385	0.169	0.116	**−**0.079	0.145
Flow slope (°)	0.032	0.460	**−0.530**	0.068	**−**0.108	**−**0.002	0.218
Flow accumulation	0.199	**−**0.487	**0.675**	0.140	0.079	0.010	0.159
Watershed size (m^2^)	0.207	**−**0.409	0.451	0.080	0.077	0.080	0.176
Air temp. (°C)	**−**0.527	0.199	0.227	**−0.480**	0.189	0.138	**−**0.356
H_2_O temp. (°C)	**−**0.426	**−**0.045	0.355	**−0.648**	0.196	0.137	**−**0.311
pH	0.222	**−0.780**	**−**0.315	**−**0.041	0.157	0.131	**−**0.173
COND (μS)	**−**0.097	**−0.841**	**−0.429**	**−**0.163	0.070	0.097	0.141
TDS (mg/L)	**−**0.089	**−0.837**	**−0.433**	**−**0.142	0.056	0.090	0.160
O_2_ (mg/L)	0.573	**−**0.131	**−**0.146	**0.657**	0.117	0.048	**−**0.240
O_2_ (%)	0.066	**−**0.310	0.112	**0.552**	**−**0.349	0.104	**−0.560**
COD (mg/L)	**−**0.371	**−**0.114	0.045	**−**0.009	**−0.679**	0.236	0.056
CaCO_3_ (mg/L)	**−**0.074	**−0.831**	**−**0.434	**−**0.154	0.084	0.122	0.064
Nitrate N (mg/L)	**−**0.553	**−**0.457	0.054	**−**0.044	**−0.440**	**−**0.456	**−**0.024
Nitrate NO_3_ (mg/L)	**−**0.460	**−**0.463	0.033	**−**0.110	**−**0.378	**−0.603**	**−**0.080
Ammonia N (mg/L)	**−0.849**	**−**0.026	**−**0.090	0.327	0.338	**−**0.141	**−**0.034
Ammonia NH_3_ (mg/L)	**−0.849**	**−**0.021	**−**0.096	0.330	0.332	**−**0.136	**−**0.032
Ammonia NH_4_ (mg/L)	**−0.850**	**−**0.024	**−**0.091	0.326	0.338	**−**0.133	**−**0.036
Chloride Cl (mg/L)	**−0.738**	0.032	0.150	0.194	**−**0.169	**0.499**	0.179
Chloride NaCl (mg/L)	**−0.739**	0.035	0.150	0.192	**−**0.170	**0.498**	0.180
Eigenvalue	4.914	4.782	2.502	1.933	1.504	1.405	1.015
Total variance %	22.334	21.735	11.374	8.785	6.838	6.388	4.615

## Discussion

Results confirmed that the physicochemical properties of individual streams were very variable in the Slovakian Tatra Mountains. This situation is affected by many factors, including bedrock structure, topography, elevation, and local climate or weather (Wohl, 2000; Sajdak et al., 2018; Radecki-Pawlik et al., 2020). Our results show that levels of basic elements in mountain streams increase with decreasing elevation, as confirmed by many authors in the region (e.g., Żelazny & Siwek 2012). If we want to know how water properties are changing in mountain streams, then we need to focus on sources like precipitation (rain, snow, fog) at the top of the mountain. There are some indications (Grodzińska-Jurczak, 1995; Rogora et al., 2006) that the physicochemical properties of mountain streams (especially at the highest elevations without glaciers) are formed from the chemical composition of precipitation in the area. This is due to fast water circulation and high resistance of bedrock, as confirmed by numerous studies regarding the acidification of the environment (e.g., Kopáček et al., 2006). The chemistry of precipitation is more likely to be altered in areas with well-developed soils (Kopáček et al., 2019). As such, climate and temperature play significant roles, and water temperature and flow rate are essential factors in the process of water mineralization (Todd et al., 2012; Manning et al., 2013). Generally, stream temperature is significantly dependant on weather conditions (including sunlight/solar radiation, heat transfer from the atmosphere), catchment characteristics, and flow rate (Żelazny et al., 2018), though flow rate is predominantly influenced by precipitation quantity. One study conducted in the Rocky Mountains (Zhi et al., 2020) observed that volume solutes from rock weathering remain stable, but solutes from soil increase, mostly in dry periods (with low flow), warm years, or during snowmelt. It is evident that physicochemical characteristics of mountain streams are different over different years or seasons (Bojarczuk et al., 2018). Available meteorological data from the station at Lomnický peak (2634 m a.s.l.) pointed to lower monthly precipitation during sample collection in 2017. Lower precipitation could lead to lower flows, affecting levels of measured compounds in streams. Therefore, our values of total dissolved solid, conductivity or CaCO_3_, and chlorides were significantly higher in 2017. Some of our computed phenomena (principal components), such as a higher accumulation of ammonias and chlorides in the alpine zone (PC1) or a higher mineralization (PC2) in alpine and sub-alpine environments, were also elevated in 2017.

Water temperature and air temperature have increased in the region over the last several decades (e.g., 0.6 °C in Danube, Pekarova et al., 2008; Melo et al., 2013). In mountainous regions, we can expect a similar effect (e.g., Hari et al., 2006; Paillex et al., 2020), impacting water quality and ecosystem health (Whitehead et al., 2009; Isaak et al., 2010; Ficklin et al., 2013). The synergic effect of temperature and low flow rate, caused by shifts in precipitation or induced earlier snowmelt and prolonged summer droughts, also decreases oxygen concentration and solubility in water, affecting self-purification processes. PC1 and PC4 partly confirm that the relationship of temperature to oxygen affected ammonia concentrations. While PC1 shows a positive trend with temperature, PC4 describes a negative trend connected more to the ammonia-oxidizing processes driven by bacteria. Dissolved oxygen enters the water environment through the air by diffusion or aeration caused by wind, waves, rapids, waterfalls, and ground water discharge, or as a plant product due to photosynthesis, respiration, and decomposition (Kannel et al., 2007). Oxygen level is also influenced by stream morphology. PC7 explains the occurrence of oxygen rich sites in lower elevations where streams are steeper, wider, and colder. Mean values of dissolved oxygen were lower in 2017, due to lower flow rate. This is concerning, as it indicates that sites rich in oxygen, necessary for some species, could be markedly affected in periods with higher temperatures and lower flows. This synergic effect could lead to migration of some species (e.g., those dependent on colder water with higher concentrations of oxygen) and push them up-river to cooler higher elevations (Hari et al., 2006). With respect to physical barriers for upward migration, and the expectation of dynamic changes to the water system in mountain environments (e.g., precipitation patterns and extreme precipitation events) caused by warming (Fort, 2015), it is questionable whether there are adequate habitats to support these species.

Rising air and water temperatures can increase biological productivity and decomposition during the growing season in oligotrophic streams (Ferreira & Chauvet 2011; Clark et al. 2021). Our data partly confirm this trend: first, through mutual positive correlation of water temperature with COD (commonly used to indirectly measure organic compounds in water) in 2010, and with compounds of nitrates, ammonias, and chlorides in 2017, and second, through PC4 and PC6, as cooler sites had higher concentrations of ammonia compounds due to inefficient ammonia oxidation processes connected to their inhibition by chlorides (Szklarek et al. 2022).

Higher levels of total dissolved solids, conductivity, and measured chemical compounds indicate that our water samples had a higher density in 2017. However, the trend of water enrichment by basic chemical compounds downstream did not vary (see Fig. 4–PC2). Krno et al. (2006) recorded lower values of conductivity in Tatra Mountain lakes that were comparable to our mean values (37 μS) measured in streams in 2010. However, in 2017, we measured conductivity levels that were almost two times higher (61 μS). On the other hand, mean pH levels were not significantly different between the years (Table 2), even though the pH values were slightly lower in 2017. Therefore, our observed differences do not contradict acidification recovery (e.g., Kopáček et al., 2006; Borg & Sundbom 2014), and slightly lower values of pH can be explained by lower precipitation amounts as well as climate development (Kopáček et al., 2019). Acidification has been discussed thoroughly by many studies focused on mountain lakes (e.g., Stuchlík et al., 2006; Kopáček et al., 2006), particularly regarding consequences to biological structure (e.g., Vranovský et al., 1994; Fott et al., 1999; Hořická et al., 2006), including water bodies located in the Tatras. Though alpine lakes have a considerable influence on the chemistry of the river basin, as confirmed by our data, it remains important to monitor changes in mountain lakes as indicators of long-term environmental change (Moser et al., 2019). In the Tatras, Kopáček et al. (2019) compared data gathered between 1992 and 2018 and noticed a climate-related increase in physical erosion, particularly in steep and scree-rich areas, which could change the trend of solute concentrations in water. Thus, higher weathering rates caused by climate change can contribute to increased leaching of nutrients.

The natural level of ammonia or nitrate in surface water is typically low (less than 1 mg/L (Wetzel, 2001)), and our measurements did not exceed this limit. Our highest value of NH_3_ was 0.35 mg/L and NH_4_ 0.37 mg/L. Higher values of ammonias and nitrates (Fig. 2A) were primarily identified in alpine zone, especially in 2017. In 2010, the highest values of nitrates were measured in lower-elevation parts of streams in developed areas, as confirmed by Holko et al. (2006) in the case of Jalovecký stream in the western part of Tatras. Ammonia and nitrate content varies throughout the day and the season. It is highest during spring and summer and lower in autumn and winter (Krno, 2006). The quantity of ammonia and nitrates in the water is also a result of the nitrification process, caused by aerobic decomposition of the microbial activity of ammonia-oxidizing bacteria and archaea (Schleper & Nicol 2010, Auguet et al., 2011). In our study, higher mean values of ammonia and nitrates were measured in sites affected by mountain lakes, but only in 2017. This might be explained by higher plant physiological activity, microbial activity, or nutrient supply from the catchment (Kaste et al., 2020). In mountain regions, the amount of ammonia in aquatic ecosystems may be influenced by human activity near mountain huts and cottages (Kangas et al., 2012). However, in areas with no human activity, it is caused by the local climate. We must realize that alpine regions are very vulnerable to environmental changes caused by climate (Beniston, 2003), and ammonia concentrations in the environment are highly dependent on temperature (Baron et al., 2009). This also confirms our data, especially from 2017 (the drier year).

## Conclusions

This work in the Tatra Mountains of Slovakia shows that the physicochemical properties of individual streams were very variable and affected by many factors, including bedrock structure, topography, elevation, and the local hydroclimate. Comparing datasets from two different years, we were able to observe common trends that may be linked. Through the mutual evaluation of data using the multivariate statistical technique of principal components, we identified seven main phenomena in selected mountain streams. The most significant was the temperature effect on ammonias and chlorides. This was followed by physicochemical properties in relation to elevation (level of basic elements in mountain streams increase with decreasing elevation). The third component described the effect of flow accumulations on total dissolved solids and CaCO_3_. The fourth component pointed to temperature driven ammonia-oxidizing processes. The fifth component explained the mutual interaction of chemical oxygen demand and nitrates with levels of ammonias in streams. The sixth component pointed to inhibition of nitrification by chlorides. The last component represented sites in higher-elevation parts of streams which are cooler and at the same time less saturated by oxygen due to stream morphology. Some of these effects had a stronger manifestation in the year 2017, which was poorer in precipitation than 2010. Based on our results, we can expect a higher degree of nutrient leaching, changes to the N-cycle in alpine areas, as well as decrease of oxygen contents. An increased focus on this issue can yield observation of these phenomena into the future, supplemented by variables that have become more accessible due to new research techniques and methods.

## Data Availability

The datasets analyzed during the current study are available from the corresponding author on reasonable request.
